# Fibromyalgia in cancer patients: a systematic review and clinical implications for integrated care

**DOI:** 10.3389/fpain.2026.1851474

**Published:** 2026-06-11

**Authors:** Melania Prete, Giuseppe Porciello, Elvira Palumbo, Sara Vitale, Maurizio Marchesini, Sabrina Bimonte, Francesco Del Prato, Arturo Cuomo, Marco Cascella, Maria Grimaldi, Natalia Russo, Assunta Luongo, Anna Crispo

**Affiliations:** 1 Epidemiology and Biostatistics Unit, Istituto Nazionale Tumori IRCCS “Fondazione G. Pascale”, Naples, Italy; 2 Division of Anesthesia and Pain Medicine, Istituto Nazionale Tumori IRCCS “Fondazione G. Pascale”, Naples, Italy; 3 Department of Medicine, Surgery and Dentistry “Scuola Medica Salernitana”, University of Salerno, Salerno, Italy

**Keywords:** fibromyalgia, integrated care, nociplastic pain, nutrition, oncology, pharmacology, physical activity, quality of life

## Abstract

**Introduction:**

In oncology, fibromyalgia (FM) is increasingly recognized as a clinically relevant comorbidity, as cancer patients frequently present overlapping symptoms such as pain, fatigue, and sleep disturbances, complicating clinical assessment and management. Moreover, anticancer treatments may trigger or exacerbate FM-like symptoms. This systematic review aimed to synthesize the available evidence on FM in oncological patients, focusing on prevalence, clinical characteristics, and its impact on pain perception, Health-Related Quality of Life (HRQoL), and treatment adherence.

**Methods:**

A comprehensive literature search was conducted in PubMed, Embase, and Scopus, including studies published from 2014 to 2024. Eligible studies, in accordance with PRISMA guidelines, included observational, cross-sectional and interventional designs involving adult patients with FM in the context of cancer or cancer-related pain. Conversely, studies not assessing FM in relation to cancer or cancer-related pain were excluded. Risk of bias was evaluated using ROBINS-I tool and the RoB 2 tool.

**Results:**

A total of 11 studies were included, encompassing heterogeneous populations and study designs, thereby precluding a quantitative synthesis of the literature. Overall, evidence suggests that FM did not increase cancer risk but significantly contributes to pain amplification, higher symptom burden, reduced HRQoL, and impaired treatment adherence. Pre-existing nociplastic pain features were associated with premature discontinuation of oncological therapies, particularly endocrine treatments. The substantial overlap between FM-related and cancer-related symptoms may represent a major diagnostic challenge in clinical practice.

**Discussion:**

This review highlights a substantial research gap, as evidence in oncology populations remains limited and is affected by moderate to high risk of bias, partly due to confounding factors. Nevertheless, FM appears to be a relevant comorbidity in oncology, warranting further investigation. High-quality longitudinal studies are needed to clarify causal associations and support evidence-based management.

**Systematic Review Registration:**

The protocol for this systematic review was retrospectively registered on the Open Science Framework (OSF; https://doi.org/10.17605/OSF.IO/X5N29).

## 1 Introduction

### 1.1 Background: fibromyalgia in oncology

Fibromyalgia (FM) is a complex chronic syndrome characterized by a wide spectrum of symptoms, including widespread musculoskeletal pain, chronic fatigue, sleep disturbances, and cognitive deficits, commonly referred to as “fibro-fog,” which can impair memory, concentration, and the ability to perform complex tasks. These symptoms can vary widely between patients, often affecting daily functioning and leading to a substantial reduction in overall physical and psychological well-being (1–3). Other frequently reported symptoms include headache, gastrointestinal disturbances, temporomandibular pain, sensory hypersensitivity, and a high prevalence of psychiatric conditions, including anxiety and depression (4–8). FM affects approximately 2%–8% of the global population, with a marked predominance in females and a higher incidence between 30 and 60 years of age. Comorbid conditions are highly prevalent, occurring in nearly 84% of patients (9–11). Although the exact cause of FM has not yet been fully elucidated, the literature suggests that the disorder arises from a complex interaction of genetic, neurobiological, and psychosocial factors (5, 12, 13).

In light of the clinical complexity of FM, the International Association for the Study of Pain (IASP) has formally recognized a third pain phenotype, termed “nociplastic pain” (14), which is distinct from nociceptive and neuropathic pain. The introduction of this category was necessitated by clinical presentations, exemplified by FM, irritable bowel syndrome (IBS), and chronic pelvic pain, where widespread pain persists despite no clear evidence of actual or threatened tissue damage or a primary lesion of the somatosensory system. Nociplastic pain is characterized by altered nociception due to the dysfunction of central pain-processing pathways. This paradigm shift emphasizes that FM is not merely a ’symptom’ but a manifestation of a centralized pain state, where the nervous system's heightened reactivity is intrinsically linked to a constellation of associated symptoms, including psychological distress, sleep disturbances, and environmental sensitivities. Integrating the concept of nociplastic pain provides a more robust framework for understanding why FM often coexists with other chronic primary pain syndromes and why it requires a specific, multimodal management strategy that differs from traditional analgesic approaches (15).

In the oncological setting, FM is receiving growing attention. On the hand, patients with FM may present with a wide range of comorbidities, including cancer (16). On the other hand, oncology patients may experience similar symptoms, either due to the disease itself or as side effects of treatment. Musculoskeletal pain, chronic fatigue, and sleep disturbances are common manifestations, often making it difficult to distinguish between cancer-related symptoms and FM-related symptoms (17–19). Cancer treatments can affect both the central and peripheral nervous systems, as well as induce chronic pain and fatigue (18). Symptom control in oncology is often challenging due to potential interactions between analgesics, supportive treatments, and anticancer therapies. Early and accurate diagnosis, together with an integrated and multidisciplinary treatment approach, is crucial to distinguish FM from cancer-related symptoms and to improve patients’ quality of life (QoL). The therapeutic approach should aim to reduce pain, fatigue, and sleep disturbances, while optimizing the overall effectiveness of oncological treatment and supporting patients’ psychophysical well-being (20–22). Finally, it should be emphasized that FM represents a significant comorbidity, potentially affecting both the patient's subjective experience of the disease and the effectiveness and management of oncological treatments (19). The prevalence and development of this condition among cancer patients can differ considerably according to cancer type, the population studied, and the diagnostic criteria applied. However, available data indicate that a substantial proportion of cancer patients present symptoms compatible with FM, either as a pre-existing condition or as a secondary phenomenon related to treatments (19). FM may be present before a cancer diagnosis, with a subsequent worsening of pain symptoms following disease onset. At the same time, certain oncological treatments, particularly chemotherapy and immunotherapy, may trigger or exacerbate FM-related symptoms, further complicating pain assessment and the selection of appropriate therapeutic strategies (19). A key element in managing cancer patients with FM is clearly distinguishing FM-related pain from cancer-related pain, as the two can overlap. FM-related pain is generally diffuse, chronic, and accompanied by fatigue, sleep disturbances, and cognitive changes, whereas cancer-related pain is often more localized or directly linked to specific tissues or lesions (23, 24). Finally, the presence of FM in cancer patients can significantly affect QoL and treatment adherence, impairing patients’ ability to tolerate oncological therapies, reducing compliance, and increasing the risk of treatment interruption or modification (19, 25, 26). Understanding the prevalence and clinical profile of FM in cancer patients is the key to designing individualized diagnostic and therapeutic approaches.

Nonetheless, the available literature on FM in oncology remains fragmented and methodologically heterogeneous, with studies differing in populations, clinical settings, diagnostic criteria, and outcome measures. To date, no comprehensive systematic synthesis has critically evaluated the available evidence on FM in oncological populations. This gap limits the ability to define its true clinical relevance and to inform evidence-based, multidisciplinary management approaches. Therefore, a systematic review is warranted to synthesize current evidence, clarify the role of FM as comorbidity in cancer patients, and identify key areas for future research.

## 2 Aim

This review aims to systematically map the existing literature on FM in oncological patients, focusing on its prevalence, clinical characteristics, and impact on pain perception, Health-Related Quality of Life (HRQoL), and treatment adherence. Particular attention is given to FM as both a pre-existing condition and a comorbidity arising during or after cancer diagnosis and treatment, as well as to the diagnostic challenges in distinguishing FM-related pain from cancer-related pain. The review also examines the influence of FM on patient-reported outcomes, including symptom burden, functional impairment, and tolerance to oncological therapies. Each outcome was defined according to the original study definitions and validated measurement instruments when available. In addition, it provides an overview of current pharmacological and non-pharmacological management strategies in oncology settings, with a focus on lifestyle interventions such as physical activity (PA) and nutrition. By synthesizing the breadth of available evidence, this systematic review aims to identify key knowledge gaps and inform future research, ultimately supporting the development of integrated, multidisciplinary, and patient-centered approaches to the management of FM in cancer patients.

## 3 Methods

### 3.1 Study design

This study is a systematic review carried out in line with the Preferred Reporting Items for Systematic Reviews and Meta-Analyses (PRISMA) guidelines (27). Following the development of the search strategy, studies were selected through a structured screening process, and relevant data were extracted. The number of records identified, assessed for eligibility, and ultimately included was carefully tracked and is presented in a PRISMA flow diagram. This systematic review was not based on a pre-established protocol and was not prospectively registered in a public database. The protocol for this systematic review was retrospectively registered on the Open Science Framework (OSF; https://doi.org/10.17605/OSF.IO/X5N29). The registration includes the review protocol, PRISMA-P checklist, and methodological details of the review process (Supplementary Tables S1–S3).

### 3.2 Search strategy

The methodological approach was designed to enable a broad exploration and mapping of the available evidence on FM in oncological settings, a field characterized by heterogeneous study designs, populations, and outcome measures. A comprehensive literature search was conducted across three major electronic databases (PubMed, Embase, and Scopus) to identify relevant studies addressing the coexistence of FM and cancer. The search strategy combined keywords and Medical Subject Headings (MeSH) related to FM, cancer, cancer-related pain, QoL, PA, exercise, and nutrition. To ensure the relevance and contemporaneity of the evidence, the search was limited to articles published in the last ten years (from 2014 to 2024) and written in English. In addition, the reference lists of selected articles were screened to identify further potentially relevant studies. Further details on full search query and translation of terms are reported in Supplementary data.

### 3.3 Eligibility criteria

Eligible studies included observational, cross-sectional, cohort, and interventional designs involving adult patients with FM in the context of cancer or cancer-related pain. Studies were considered relevant if they addressed epidemiology, clinical characteristics, pain mechanisms, QoL, or the therapeutic management of FM within oncological populations. Conversely, studies involving adults without FM, pediatric or adolescent populations as well as patients with FM not evaluated in the context of cancer or cancer-related pain were excluded. Animal and *in vitro* models were excluded. Reviews, editorials, case reports, conference abstracts, and studies not specifically focusing on the relationship between FM and cancer were excluded.

### 3.4 Study selection and synthesis of results

The systematic literature search and study selection were performed by two independent authors and any incongruity was resolved through a discussion and reaching consensus. All retrieved records were imported into Zotero reference management software, where duplicates were identified and removed. The remaining records were screened for relevance based on titles and abstracts, followed by full-text evaluation of potentially eligible articles. Study selection was conducted according to predefined inclusion and exclusion criteria, aiming to capture the full breadth of available evidence rather than limiting inclusion to specific study designs or outcomes. For each included study, relevant data were extracted using a standardized charting approach, capturing study characteristics, population features, study design, outcomes of interest, and key findings. No imputations for missing data were performed. Two investigators extracted the data independently, and any incongruity was resolved through a discussion by reaching consensus. Given the limited number of eligible studies and the heterogeneity in the outcomes investigated, a quantitative synthesis of results was not performed. Key findings were organized by outcome domain to facilitate comparison across studies.

### 3.5 Risk of bias assessment

**Figure 1:**
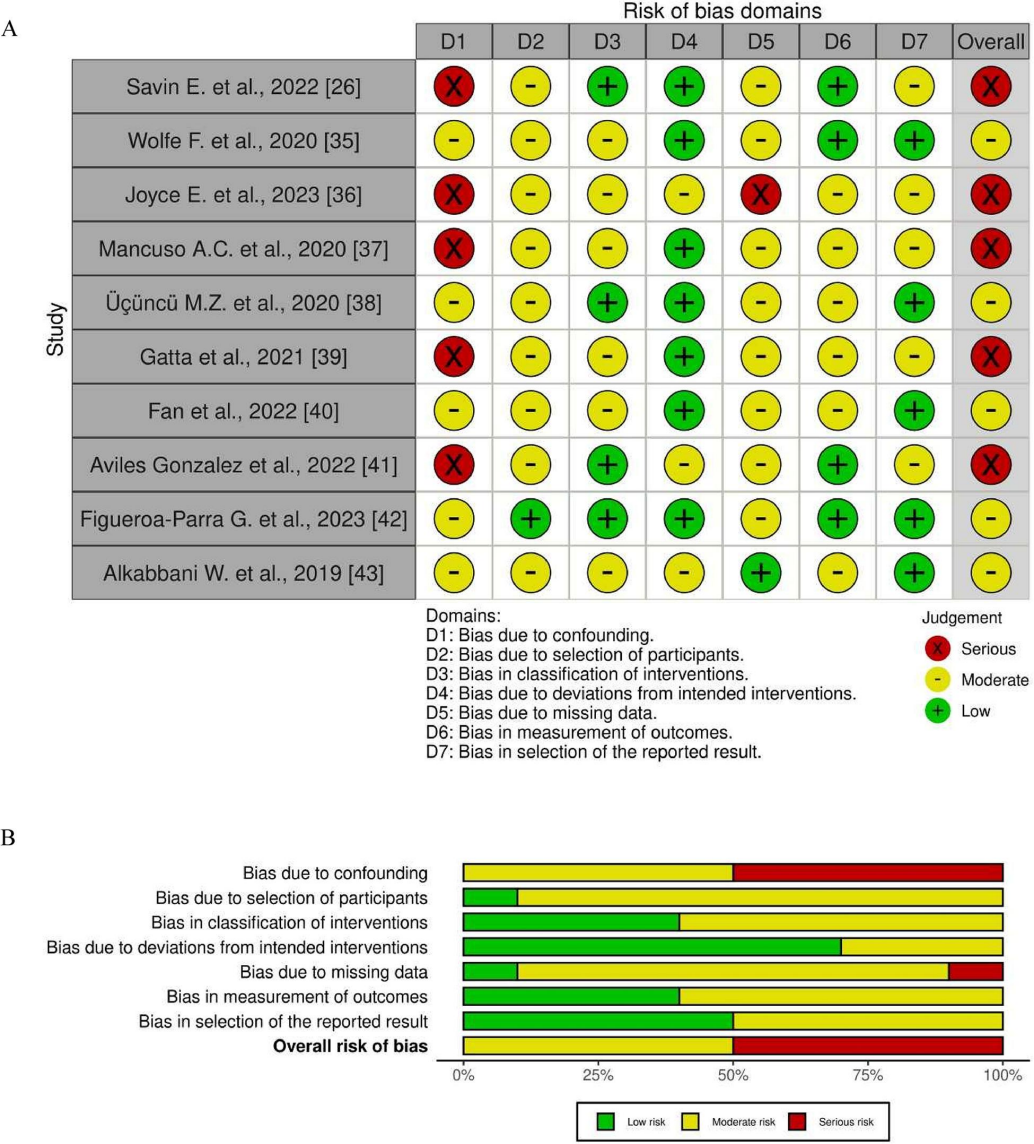
Risk of bias assessment for observational studies (ROBINS-I). **(A)** Traffic light plot summarizing the risk of bias across domains for the 10 included observational studies (retrospective and prospective cohort designs), assessed using the ROBINS-I tool. **(B)** Weighted bar plot showing the overall percentage distribution of studies across risk of bias categories (low, moderate, serious).

**Figure 2:**
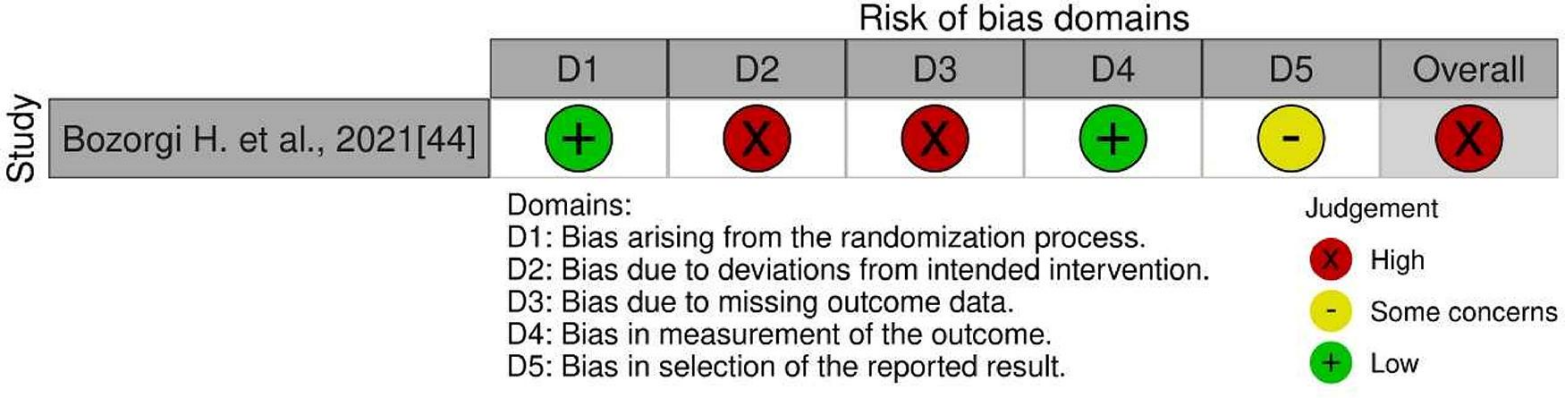
Risk of bias assessment for randomized study (RoB 2). Traffic light plot summarizing the risk of bias across domains for the included randomized study, assessed using the RoB 2 tool.

For the assessment of risk of bias across the included studies, we applied the ROBINS-I tool (28) for 10 observational studies and the RoB 2 tool (29) for the single randomized trial. For ROBINS-I, we evaluated bias across seven domains: (1) confounding, (2) selection of participants, (3) classification of interventions, (4) deviations from intended interventions, (5) missing data, (6) measurement of outcomes, and (7) selection of the reported result. For RoB 2, the following domains were considered: (1) bias arising from the randomization process, (2) bias due to deviations from intended interventions, (3) bias due to missing outcome data, (4) bias in measurement of the outcome, and (5) bias in selection of the reported result. Each domain was independently assessed by two reviewers, with disagreements resolved by consensus. The results are summarized in a table using color-coded indicators to reflect the level of risk for each domain and each study, allowing a clear and rapid visualization of potential biases and supporting a transparent appraisal of the overall quality of evidence. We employed the ROBVIS tool (30) to generate graphical representations of the assessments, including traffic-light and summary plots (Figures 1A, B, 2), facilitating a visual comparison of risk across studies and domains.

## 4 Results

### 4.1 Characteristics of included studies

**Figure 3:**
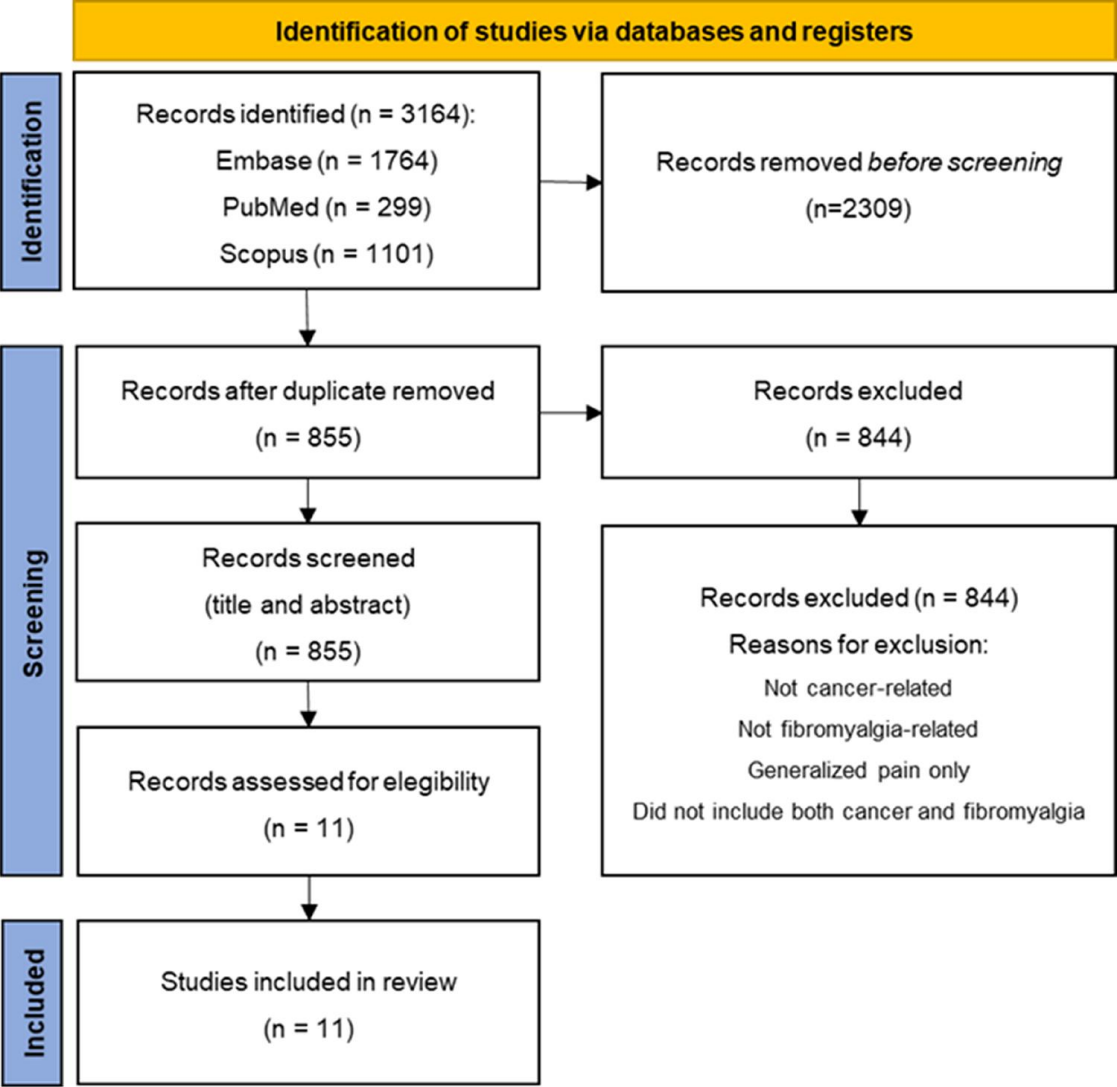
Preferred reporting items for systematic reviews and meta-analyses 2020 (PRISMA 2020) flow diagram. Study selection process: 3,164 records identified, 2,309 removed before screening, 855 screened, 844 excluded, and 11 studies included in the qualitative synthesis.

The database search identified 3,164 records (EMBASE: *n* = 1,764; PubMed: *n* = 299; Scopus: *n* = 1,101). Before screening, 2,309 records were removed as duplicates or ineligible. After duplicate removal, 855 records were screened based on title and abstract. Of these, 844 records were excluded for the following reasons: not cancer-related, not FM-related, focused on generalized pain only, or did not include both cancer and FM. Eleven full-text articles were assessed for eligibility, all of which met the inclusion criteria and were included in the final qualitative synthesis. The study selection process is summarized in Figure 3. Eleven studies were included in this review, encompassing diverse countries, study designs, and populations. Most studies were conducted in North America (USA and Canada), followed by Europe (Italy, England, Israel, Turkey) and Asia (China, Iran). Study designs included cross-sectional observational studies, retrospective and prospective cohort studies, and one randomized controlled trial, reflecting a broad methodological spectrum. Sample sizes varied widely, ranging from 151 to 64,991 participants, with most studies including both male and female patients: some focused exclusively on female populations, particularly in the context of breast cancer screening or fertility. Mean participant ages ranged from 39.9 to 63.1 years, reflecting adult and older adult populations relevant to cancer care. FM was defined according to established clinical criteria (e.g., ACR criteria) or assessed using validated screening tools, while outcomes of interest included prevalence of FM, association with pain and nociplastic symptoms, HRQoL, treatment adherence, and patient-reported satisfaction with therapy. Analytical approaches varied from logistic and Cox regression models to ANOVA, Kaplan–Meier survival analysis, and randomized crossover interventions. Key findings consistently highlighted the complex interplay between FM and cancer-related conditions. Some studies reported high co-occurrence of FM with gastrointestinal disorders, IBS, and chronic pain, while others emphasized the negative impact of FM on HRQoL, pain severity, and treatment adherence. Notably, interventions such as crocin were investigated for chemotherapy-induced neuropathic pain, suggesting potential therapeutic implications for FM in oncology populations. Overall, the included studies demonstrate significant heterogeneity in study populations, methodologies, and outcome measures, underscoring the need for a comprehensive synthesis to guide future research and clinical practice. The main characteristics and findings of the included studies are summarized in Supplementary Table S4 (S4).

### 4.2 FM, comorbidity, and cancer risk

Large-scale epidemiological analyses conducted by Savin et al. (19) evaluated the association between FM and both benign and malignant gastrointestinal conditions in a large patient cohort. The study demonstrated a strong association between FM and several benign gastrointestinal disorders, including IBS, gastroesophageal reflux disease, and inflammatory bowel diseases. However, no significant differences were observed in the prevalence of gastrointestinal malignancies between patients with FM and matched controls. These findings suggest that FM is not associated with an increased risk of cancer but rather reflects a heightened somatic and multisystem vulnerability.

Consistent results were also reported by Mancuso et al. (31), who analyzed a cohort of U.S. women veterans and found a higher prevalence of FM among individuals with a history of infertility, along with poorer physical HRQoL. Although an initial unadjusted association between infertility and cancer was observed, this relationship was no longer significant after adjustment for major confounding factors, supporting the interpretation of FM as a marker of systemic health burden rather than a direct cancer risk factor.

### 4.3 Symptom severity and pain amplification

The study by Wolfe et al. (32) provided important insights into FM as a quantitative expression of physical comorbidity. The authors showed that an increasing number of comorbid conditions were associated with a progressive rise in widespread pain indices, symptom severity, and polysymptomatic distress. Although not specifically focused on oncological populations, these findings provide a robust interpretative framework for understanding the amplification of pain and symptom burden in cancer patients with FM or nociplastic pain features. In the gastroenterological setting, Üçüncü et al. (33) reported a substantial overlap between FM and IBS, with FM present in more than 25% of IBS patients. In this study, generalized pain emerged as the strongest predictor of FM, along with functional impairment, psychological stress, and cancer-related anxiety, highlighting the central role of pain amplification in the clinical phenotype.

### 4.4 Impact of FM in oncological settings

Direct evidence regarding the impact of FM in oncology derives from Joyce et al. (34), who investigated the role of preoperative nociplastic pain in patients with breast cancer undergoing adjuvant endocrine therapy. Higher FM survey scores were independently associated with an increased risk of premature discontinuation of endocrine treatment, regardless of other clinical and demographic factors. This study provides clear evidence that FM and nociplastic pain can negatively influence treatment tolerance and adherence in oncology patients.

Within a cancer screening context, Gatta et al. (35) observed that a substantial proportion of women reporting severe pain during mammographic examination were subsequently diagnosed with FM. Although therapeutic outcomes were not directly assessed, these findings suggest that exaggerated pain responses during standard diagnostic procedures may serve as a clinical indicator of underlying nociplastic pain mechanisms.

### 4.5 HRQoL and perceived disease burden

The impact of FM on HRQoL was clearly demonstrated in the study by Gonzalez et al. (36); the authors compared patients with solid cancer to healthy controls and to patients with other chronic conditions. In this analysis, FM was associated with a greater impairment in HRQoL than solid cancer, while depressive episodes emerged as the primary determinant of reduced HRQoL, independent of cancer severity. Real-world data from Fan et al. (37) further revealed low diagnostic rates and suboptimal treatment satisfaction among patients with cancer-related pain and FM, highlighting substantial gaps in the recognition and management of complex pain conditions within these populations.

### 4.6 Therapeutic implications

Finally, studies investigating pain management strategies highlighted the significant influence of FM on therapeutic patterns. Figueroa-Parra et al. (38) showed that among patients with systemic lupus erythematosus (SLE), FM was the strongest predictor of long-term opioid therapy, surpassing the contribution of other comorbidities and organ manifestations. Complementary findings from Alkabbani et al. (39) indicated that FM was associated with longer persistence in the use of prescribed cannabinoid medications, whereas a cancer diagnosis predicted earlier treatment discontinuation. In parallel, the clinical trial conducted by Bozorgi et al. (40) provided preliminary evidence supporting a mechanism-based approach to pain management, demonstrating that crocin significantly reduced symptoms of chemotherapy-induced peripheral neuropathy with a favorable safety profile.

## 5 Discussion

Across different study designs and populations, the evidence indicates no association between FM and an increased risk of cancer or cancer-related mortality. Large population-based studies have not observed a higher prevalence of cancer among individuals with FM, suggesting no association between FM and increased cancer risk (19, 31). On the other hand, convergent evidence on FM indicates a possible role in amplifying pain perception and symptom severity, particularly in medically complex and oncological populations. Wolfe et al. and Üçüncü et al. show that generalized pain and comorbidities are key features of the FM phenotype. This provides a conceptual framework to understand the higher symptom burden observed in cancer patients with nociplastic pain features (32, 33). Within oncology-specific contexts, Joyce et al. provided direct evidence that FM-related nociplastic pain may adversely affect treatment tolerance. In this study, it independently predicts early discontinuation of adjuvant endocrine therapy in patients with breast cancer (34). These findings are consistent with Gatta et al., who observed that exaggerated pain responses during routine diagnostic procedures may indicate underlying nociplastic mechanisms (35).

HRQoL is strongly impacted by FM, sometimes to a degree comparable with or exceeding that seen in solid cancers. Depressive symptoms remain a key determinant, independent of the severity of the underlying malignancy. Real-world data from Fan et al. revealed significant gaps in the diagnosis and management of both FM and cancer-related pain. Low diagnostic rates and poor treatment satisfaction highlight the need for better pain recognition and tailored management strategies in oncology care (37). FM influences analgesic use and long-term treatment persistence. Evidence from Figueroa-Parra et al. (38) and Alkabbani et al. (39) indicates consistent associations with prolonged opioid and cannabinoid use. At the same time, cancer-related pain alone is not a predictor of continued use of centrally acting analgesics. Bozorgi et al. highlight the potential benefits of mechanism-based approaches for cancer-related neuropathic pain, emphasizing that treatment strategies should target the underlying pain mechanisms rather than relying on a uniform analgesic regimen (40).

Although studies examining both FM and cancer are limited, they provide valuable insights. Most included studies were observational or cross-sectional, preventing the establishment of causal or temporal relationships between FM, cancer progression, and treatment outcomes. Although these studies reveal important associations, they do not clarify the direction of effects. Consequently, reliance on self-reported diagnoses and symptom measures may have introduced misclassification or reporting bias. Furthermore, some analyses were conducted in non-oncological populations and extrapolated to cancer settings, potentially limiting their applicability. Small sample sizes in certain studies and the absence of well-matched control groups in others further constrain the generalizability of the findings. Moreover, heterogeneity in diagnostic criteria for FM and variability in pain assessment tools across studies represent additional sources of methodological inconsistency. Finally, the risk-of-bias assessment indicated a moderate to high risk of bias, particularly due to confounding factors. This further supports the hypothesis that, based on the current state of knowledge, it is not possible to establish a causal relationship or determine the directionality of the associations reported in the studies. Consequently, this analysis reinforces the need for high-quality studies with greater methodological rigor and improved study design. Nonetheless, given the paucity of studies examining the interrelationship between FM and cancer, as well as the complexity of the topic, it is plausible that the effect of confounding factors could not be adequately distinct. The main strength of this review lies in its integrative and multidimensional approach. By synthesizing evidence from epidemiological, clinical, real-world, and interventional studies, this review offers a comprehensive perspective on FM as a plausible modifier of clinical experience in oncology. The explicit focus on patient-reported outcomes, pain mechanisms, lifestyle factors, and therapeutic implications provides clinically relevant insights that extend beyond traditional disease-centered models. Furthermore, the inclusion of studies addressing nutrition, PA, and QoL allows for a broader understanding of FM within supportive and integrative oncology frameworks. FM in cancer patients remains largely underexplored. Longitudinal studies are needed to track symptom onset and progression, identify risk factors, and clarify the biological mechanisms linking cancer, treatments, and FM, including inflammatory, neuroendocrine, and central nervous system alterations. Implementing multidimensional interventions that combine pharmacological and non-pharmacological strategies, such as individualized exercise, nutritional support, rehabilitation, and psychosocial care, is crucial to assess their impact on pain, fatigue, stiffness, sleep, and overall QoL. Nevertheless, the limited evidence currently available on the impact of FM in cancer patients highlights the need for further investigation to better investigate these aspects through additional studies. Indeed, based on the current state of knowledge, it is not yet possible to provide a synthesis on available, lifestyle-based interventions tailored for oncologic patients with FM with a high degree of accuracy. Moreover, the evaluation of HRQoL in cancer patients who also present with FM remains largely underexplored. Consequently, drawing on the available evidence that has investigated the impact of lifestyle-related factors and interventions in FM and cancer patients as separate clinical entities, we attempted to outline potential clinical implications, although these should currently be considered highly preliminary and limited. Developing evidence-based guidelines for this population could standardize care, promote interdisciplinary collaboration, and integrate systematic QoL assessments as a core component of patient-centered management. In summary, the current evidence highlights that FM, while not increasing cancer risk, represents a clinically meaningful comorbidity in oncology. It may substantially affect symptom burden, QoL, and therapeutic trajectories, with relevance for pain management and treatment adherence. Taken together, these findings suggest a multifaceted impact of FM in cancer patients, hinting at clinical and integrative considerations that require further longitudinal investigations.

## 6 Implications for clinical management

**Figure 4:**
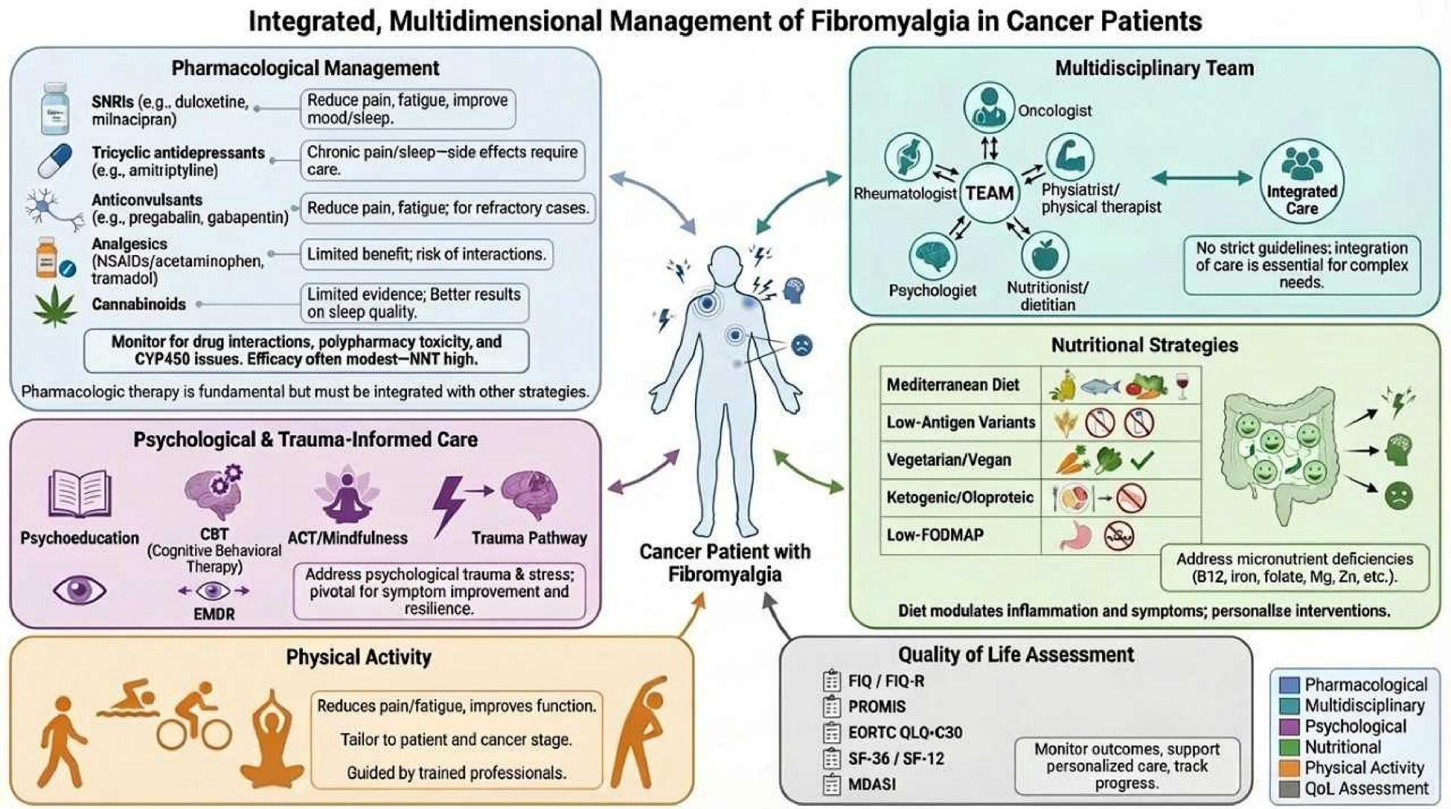
Multidisciplinary and personalized framework for cancer patient management. Schematic overview of an integrated approach for cancer patient management, highlighting multidisciplinary care, personalized treatment strategies, and supportive services.

The management of FM, particularly in cancer patients, requires a multimodal approach that targets pain, fatigue, sleep disturbances, and psychological distress through coordinated pharmacological and non-pharmacological interventions (Figure 4).

### 6.1 Pharmacological management in cancer patients with FM

Pharmacological management represents an important component of a multimodal approach to cancer patients with FM. This therapy is aimed at alleviating pain, stiffness, fatigue, and sleep disturbances, and should always be considered within the context of ongoing cancer treatments, taking into account potential drug–drug interactions and individual tolerability (43). Antidepressants, particularly serotonin–norepinephrine reuptake inhibitors (SNRIs) such as duloxetine and milnacipran (FDA-approved for FM in the US, but not received approval from the EMA for this indication in Europe), have been shown to reduce pain and fatigue while improving sleep quality and mood in patients with FM. Tricyclic antidepressants, including amitriptyline, can also be beneficial, especially for patients experiencing chronic pain and sleep disturbances; however, their potential side effects require careful consideration. These medications are currently considered first-line pharmacological options for FM and may be particularly useful for oncology patients who also present with depressive or anxiety symptoms (41, 42, 44). Among anticonvulsants, pregabalin is the most extensively studied agent in FM and has been shown to significantly reduce pain symptoms and fatigue, whereas evidence-supporting gabapentin is more limited. These medications modulate central pain sensitization and may be combined with antidepressants in more refractory cases, with careful attention to potential adverse effects such as somnolence and dizziness (45, 46). Conventional analgesics have a limited role in FM management. Nonsteroidal anti-inflammatory drugs (NSAIDs) and acetaminophen provide only modest benefit, while agents such as tramadol may be useful in selected cases due to their dual opioid and neurotransmitter-modulating mechanisms. However, their use should be carefully evaluated, particularly in oncology patients who are frequently exposed to multiple concomitant medications (47, 48). In recent years, there has been growing interest in the endocannabinoid system and the potential role of cannabinoids in the treatment of FM. Through CB1 and CB2 receptors, the endocannabinoid system regulates pain, mood, inflammation, and stress, which are all highly relevant to FM (49). However, the available clinical evidence remains limited and generally of low quality, as most studies are small-scale trials with short follow-up periods and heterogeneous methodologies (50). Some reviews report modest benefits in terms of pain reduction and QoL, whereas others do not identify significant effects. Overall, robust evidence supporting the clinical use of cannabinoids in FM is still lacking, although more recent findings suggest that short-term improvements may be observed in specific contexts (51). Pharmacological therapy for FM in oncology is significantly limited by modest efficacy and a high Number Needed to Treat (NNT) (41, 52). The management is further complicated by the risk of cytochrome P450 interactions with anticancer agents, the potential for opioid-induced immunosuppression, and the cumulative toxicity of polypharmacy. These constraints highlight the clinical necessity of moving beyond a purely analgesic model, providing the rationale for prioritizing non-pharmacological interventions, such as nutrition and PA, within an integrated care framework (53–55).

### 6.2 Multidisciplinary assessment

Patients experiencing both cancer and FM-related symptoms require a multidisciplinary approach that combines clinical care with supportive strategies to address their complex, multifaceted needs (56). Oncologists play a central role in cancer care, overseeing pharmacological treatments, while rheumatologists are crucial for diagnosing and managing FM symptoms, including widespread pain, stiffness, and fatigue. Physiatrists and physical therapists contribute by developing individualized exercise programs to enhance functional capacity, mobility, and tolerance to cancer treatments (57). Nutritionists and dietitians deliver targeted dietary interventions to support fatigue management and maintain adequate nutritional status during cancer treatment, while psychologists and other mental health professionals help address stress, anxiety, and depression, which are frequently intensified by the coexistence of FM and cancer. Currently, no specific guidelines provide an integrated approach to FM in cancer patients; nevertheless, existing evidence highlights the value of personalized, multidisciplinary management protocols. Integrating non-pharmacological interventions, such as physical exercise, physiotherapy, psychological support, and nutritional assessment with tailored dietary strategies, into cancer care pathways, along with the active involvement of a multidisciplinary team, is essential for comprehensive patient management.

### 6.3 Psychological support and trauma-informed care in FM and cancer

The integration of structured psychological support is a cornerstone in the management of FM, particularly within the oncological population, where the “top-down” mechanisms of pain amplification are most prominent (58). Current evidence underscores that adverse life events and psychological trauma, especially childhood maltreatment such as physical, emotional, or sexual abuse and neglect, are significant risk factors for the onset and maintenance of FM. These early traumatic experiences can lead to long-term alterations in the developing nociceptive system and dysregulation of the hypothalamic-pituitary-adrenal (HPA) axis, favoring a state where the central nervous system (CNS) acts as the primary generator of pain (59, 60). In this clinical framework, a cancer diagnosis represents one of the most severe and stressful life events an individual can encounter, potentially serving as the decisive trigger for the “unmasking” of a latent FM profile or the acute exacerbation of pre-existing nociplastic symptoms.

The overwhelming emotional burden of oncological disease can deplete a patient's coping resources, intensifying the perception of pain and associated symptoms through the further breakdown of descending inhibitory pathways. Consequently, an integrated multidisciplinary approach must prioritize evidence-based psychological interventions: Psychoeducation, Cognitive-Behavioral Therapy (CBT), Acceptance and Commitment Therapy (ACT) and Mindfulness, and Eye Movement Desensitization and Reprocessing (EMDR). Psychoeducation represents an essential first step to validate the patient's suffering, dispel misconceptions about the “invisible” nature of FM, and foster a realistic outlook on treatment. Cognitive-behavioral therapy (CBT) is widely recognized for its effectiveness in reducing pain catastrophizing and promoting more adaptive coping strategies. Third-wave approaches, such as acceptance and commitment therapy (ACT) and mindfulness, further support patients in improving pain acceptance and emotional regulation, thereby reducing psychological reactivity to chronic discomfort. In patients with a documented history of trauma, eye movement desensitization and reprocessing (EMDR) may be particularly beneficial, as it facilitates the reprocessing of traumatic memories that can contribute to persistent physiological arousal and sensitization. The implementation of these psychological strategies within an integrated care pathway is crucial not only for alleviating the symptom burden but also for enhancing the overall resilience and QoL of cancer patients facing the complexities of nociplastic pain.

### 6.4 Dietary intervention in FM and cancer patients

Nutritional assessment in patients with FM, cancer, or both conditions concurrently is essential. These populations often experience altered dietary intake, changes in body composition, and risks of malnutrition, both due to insufficient or excessive intake, which can impact clinical symptoms, QoL, and treatment outcomes. In oncological patients with FM, diet represents a supportive yet essential component of symptom management, helping to alleviate chronic pain, fatigue, and inflammation. These patients have distinct nutritional needs due to the combined effects of cancer, its treatments, and overlapping FM symptoms. The nutritional approach in this population aims to reduce inflammation and oxidative stress, support immune function, maintain energy levels and muscle mass, minimize pain and gastrointestinal complications, and prevent nutrient deficiencies. Evidence suggests that the anti-inflammatory properties of the Mediterranean diet (MedDiet) may help mitigate this inflammatory burden, improving pain management and overall patient well-being (61). Greater adherence to the MedDiet results in lower pain intensity and other FM-related symptoms in non-oncology patients with FM (62, 63). The MedDiet is rich in antioxidants and bioactive compounds, largely due to its high intake of fruits, vegetables, extra virgin olive oil, nuts, and whole grains, along with moderate wine consumption and herbal infusions such as sage, mint, thyme, chamomile, and anise. In addition, key components such as olive oil, fish, and nuts provide beneficial fatty acids, including monounsaturated fats and omega-3s, which help modulate inflammatory pathways, promote resolution processes, and support neural function by preserving cell membrane integrity (64–66). Within the MedDiet, foods such as fatty fish (e.g., sardines and anchovies), dairy products, nuts, and seeds are important sources of vitamin D (67). Beyond its role in bone metabolism (68, 69) and calcium homeostasis (70), it has also been shown to exert anti-inflammatory effects and to modulate immune function and cellular growth (71). The low-antigen diet avoids foods with high antigenic properties and promotes foods with anti-inflammatory properties. The MedDiet promotes low-antigen foods through increased consumption of vegetables, fruits, whole grains, legumes, olive oil, and fish, and a lower consumption of animal products (64). Mild modifications of the MedD, such as the exclusion of common allergens like gluten and dairy products, can further enhance its low-antigenic profile (72, 73). Evidence from non-oncology populations, indicates that a gluten-free diet may reduce pain and fatigue in patients with FM, particularly in those with non-celiac gluten sensitivity. Symptom improvement has been reported after short-term gluten-free interventions, with recurrence following gluten reintroduction (74). In the context of FM and cancer, gut bacteria appear to play a significant role in symptom modulation and disease mechanisms. Emerging evidence suggests that alterations in the intestinal microbiota may contribute to chronic pain, including FM, with studies reporting both quantitative changes, such as altered levels of *Coprococcus, Lactobacillus*, and *Bifidobacterium*, and qualitative alterations, including small intestinal bacterial overgrowth. These findings highlight the potential interplay between microbiota, immune responses, and symptom burden in patients with FM, which may be particularly relevant in oncological settings where systemic inflammation and treatment-related effects are common (75). In FM, gut microbiome alterations extend beyond bacterial composition to changes in microbial metabolic activity, including the production of metabolites that may influence pain and fatigue (75, 76). Increased intestinal permeability may allow pro-nociceptive molecules, such as glutamate, into circulation while limiting beneficial precursors like tryptophan (75). These bacteria may influence pain, fatigue, mood, and other symptoms by allowing short-chain fatty acids, bile acids, neurotransmitters, and bacterial antigens to enter the circulation (76). Although differences in microbiota composition between patients and healthy individuals have been reported, their functional role remains unclear. Preliminary evidence suggests that restoring a healthy microbiota through transplantation may reduce pain and improve QoL (77). Dietary modulation of the gut microbiota is a potential strategy to alleviate symptoms in FM and cancer. Fermentable Oligosaccharides, Disaccharides, Monosaccharides, and Polyols (Low-FODMAP) dietary regimen has been shown to reduce gastrointestinal symptoms, pain, fatigue, and sleep disturbances, highlighting the role of the gut-brain axis (78, 79) in non-oncology patients with FM. Improvements in pain and overall clinical features have been reported, suggesting it may complement pharmacological therapy (80, 81). In cancer, particularly pelvic malignancies, preliminary studies suggest that a low-FODMAP diet may reduce radiotherapy-induced gastrointestinal symptoms, including rectal gas and diarrhea, and improve QoL. However, evidence is limited, and larger trials are needed to confirm efficacy, safety, and implementation strategies (82). Observational studies showed that vegetarian diets have been associated with improvements in certain FM symptoms, potentially due to their lower fat and protein content combined with higher intakes of dietary fiber, vitamin C, beta-carotene, essential minerals such as magnesium, potassium, zinc, and selenium, and a broad spectrum of antioxidant compounds (83). Dietary patterns such as vegan or vegetarian diets may also provide benefits for cancer survivors experiencing fatigue, likely through their anti-inflammatory and antioxidant properties. Compared with non-vegetarians, vegan diets are associated with a more favorable fatty acid profile and higher intakes of polyphenols and specific antioxidant compounds (84). Other dietary strategies that may help alleviate FM symptoms include oloproteic and ketogenic protocols. Very low-calorie ketogenic diets (VLCKD) have shown effectiveness in various inflammatory conditions, including metabolic, neurological, autoimmune disorders, and some cancers (85, 86). This study demonstrated that an oloproteic variant of a very low-calorie ketogenic diet (VLCKD) effectively improved symptoms in 45 female FM patients over 45 days. Patients following the oloproteic diet, with minimal carbohydrates and protein and fat tailored to BMI, showed greater symptom improvement than those on a moderate low-glycemic-index (LOGI) diet (85). Dietary alterations may result in significant imbalances in essential micronutrient intake. In patients with FM, deficiencies have been reported in key vitamins and minerals, including iron and vitamin B12 (87, 88); also, in non-anemic FM patients iron deficiency (<50 ng/mL) is commonly observed. In the study by Munipalli et al., an association between vitamin B12 levels and fatigue in FM patients was observed. This represents the first report of such a link, with fatigue being common among FM patients with B12 deficiency, independent of whether the cutoff was 400 or 350 ng/L (89). Iron deficiency in FM is exacerbated by the use of analgesics, which can also reduce the levels of vitamins C and K. This deficiency is particularly relevant because iron is essential for the metabolism of serotonin and dopamine, neurotransmitters that play a key role in the pathophysiology of FM (90, 91). In addition to B12 and iron, deficiencies in vitamins A, E, folate, selenium, calcium, zinc (Zn), and magnesium (Mg) have been associated with increased oxidative stress and reduced antioxidant capacity in patients with FM (90, 91). Notably, Batista et al. reported that higher vitamin E intake was associated with enhanced vasodilation and improvement of vasomotor pain, which is linked to the vasomotor dysregulation and muscle hypoperfusion observed in FM (90). Among other vitamins, vitamin B6 intake was also associated with lower pain intensity, further emphasizing the role of micronutrients in modulating symptoms in women with FM (92). Therefore, even in cancer, micronutrient deficiencies are common due to factors such as the disease itself, treatment-related side effects, reduced dietary intake, and malabsorption. Addressing these deficiencies through dietary interventions or supplementation may improve patient outcomes by supporting immune function, reducing treatment-related complications, and enhancing overall QoL (93). Consequently, a unified dietary strategy capable of preserving patients facing either or both of these conditions becomes essential. Nonetheless, the abovementioned evidence has been derived from studies conduct on FM and cancer as distinct entities, whereas evidence specifically focusing on populations affected by both conditions simultaneously remains extremely scarce. Consequently, further studies are warranted to investigate the role of dietary interventions in modulating the coexistence of both FM- and cancer-related symptoms. Such an approach may contribute to a better understanding of the potential of diet in the management of this complex symptom burden.

### 6.5 PA as a therapeutic strategy

PA is widely recognized as a therapeutic component in the management of FM, owing to its beneficial effects on pain, fatigue, sleep quality, physical function, and overall psychological well-being. Evidence consistently shows that light aerobic activities such as walking, stationary cycling, or low-intensity swimming aim to improve muscular endurance and cardiorespiratory capacity, reducing pain perception. Targeted stretching and mind–body disciplines (yoga, tai chi) enhance flexibility, balance, stress regulation, and QoL (94–98). Recent evidence in oncology highlights that maintaining PA for about one year after first-line treatment is a key window to reduce fatigue. Integrating recovery phase-specific activity, such as walking, into survivorship care, together with addressing micronutrient deficiencies, underscores the importance of comprehensive lifestyle strategies to preserve health and QoL in patients with chronic conditions or cancer (99). Structured exercise programs have been associated with reductions in anxiety, depression, and fatigue, as well as improvements in physical function and QoL during and after oncological therapies (100, 101). Emerging evidence indicates that exercise may represent not only a supportive intervention but also a modifiable prognostic factor. Scientific societies in oncology are progressively incorporating PA recommendations into clinical guidelines, emphasizing the need for tailored and supervised interventions that account for tumor characteristics, disease stage, and patient-specific factors. In this context, the implementation of structured programs to mitigate cancer- and FM-related side effects represents a crucial strategy to enhance QoL in these patients, guided by trained professionals such as physiotherapists, who can provide tailored interventions. Nonetheless, although current evidence support the need for tailored physical exercise programs in oncology patients and concomitant FM, data regarding their beneficial effects remain extremely scarce. Consequently, further studies are warranted to evaluate the effects of physical exercise in this complex population of cancer patients, with the aim of assessing its efficacy, safety, and optimal duration in order to improve symptom burden and QoL.

### 6.6 HRQoL in oncological and FM patients

HRQoL in oncological patients with FM is markedly impaired due to the overlap between cancer-related symptoms and those of FM. Chronic widespread pain, muscle stiffness, persistent fatigue, and sleep disturbances combine with treatment-related side effects such as nausea, neuropathy, and muscle weakness, leading to a significant negative impact on daily activities, work performance, and social interactions. Moreover, anxiety, depression, and emotional distress may be exacerbated by the continuous perception of physical discomfort, leading to reduced psychological resilience and diminished social participation (102–104). Validated instruments, such as questionnaires, represent a key component in the clinical management of these patients. Disease-specific tools, such as the Fibromyalgia Impact Questionnaire (FIQ) (105) for FM, as well as generic tools for oncological populations, such as the EORTC QLQ-C30 (106), enable standardized assessment of pain, fatigue, emotional well-being, and physical functioning. Regular use of these instruments allows early detection of symptom changes and helps evaluate the effectiveness of both pharmacological and non-pharmacological interventions. Assessing QoL supports improvements in physical, emotional, and social well-being, encourages patient engagement in treatment decisions, and enhances satisfaction and adherence to oncological and rehabilitative care (107). In managing oncological patients with FM, systematically using validated QoL assessment tools is key to tailoring interventions, tracking treatment outcomes, and ultimately improving overall patient well-being. The main characteristics of these questionnaires are shown in Supplementary Table S5 (S5).

## 7 Conclusions

In cancer patients, FM represents an emerging clinical challenge with significant implications for diagnosis, management, and QoL. Early recognition, with careful differentiation from cancer-related pain, is essential to guide appropriate interventions. Integrated, patient-centered management that addresses both physical and psychological aspects, potentially including tailored rehabilitation, exercise programs, nutritional support, and psychosocial interventions, can substantially improve QoL. Nonetheless, research involving oncology populations remains scarce, and available evidence is largely characterized by a moderate to high risk of bias, partly attributable to confounding factors. Both these aspects limit the strength of our conclusions. Consequently, implications for clinical practice should be interpreted with caution, as a greater number of well-designed studies are needed to elucidate the impact of FM in cancer patients. Moreover, future research should also focus on understanding the onset and mechanisms of FM in oncology patients, as well as testing combined interventions to improve functional outcomes, enhance resilience, and support patients in coping with the burden of disease. Future investigations should additionally move beyond predominantly descriptive and multidisciplinary approaches toward large-scale, data-driven research programs aimed at clarifying the relationship between FM and cancer. The integration of nationwide Electronic Health Records (EHRs), oncology registries, and longitudinal real-world databases may provide more robust epidemiological and mechanistic insights into the association between FM, cancer-related pain, and oncological outcomes. Similar approaches have already demonstrated promising results in autoimmune and inflammatory diseases, supporting the potential value of population-based cohort studies and big-data methodologies in this field (108, 109).

## Data Availability

The original contributions presented in the study are included in the article/Supplementary Material, further inquiries can be directed to the corresponding author.
