# Supplementary material for: Fibromyalgia in cancer patients: a systematic review and clinical implications for integrated care
Source: Front Pain Res (Lausanne). 2026 Jun 11;7:1851474. doi: 10.3389/fpain.2026.1851474 (PMC13294063; doi:10.3389/fpain.2026.1851474)
Supplement: Supplementary file 3 [file Table3.docx]

**Review title**

Fibromyalgia in Cancer Patients: A Systematic Review and Clinical Implications for Integrated Care

**Review type**

Systematic review

**Condition or domain being studied**

*Cancer Pain; Cancer; Fibromyalgia; Multidisciplinary comprehensive care management ; Nutritional Support; Psychological Assessment; Symptom Management; Cannabinoids*

**Rationale for the review**

Available literature on FM in oncology remains fragmented and methodologically heterogeneous, with studies differing in populations, clinical settings, diagnostic criteria, and outcome measures. To date, no comprehensive systematic synthesis has critically evaluated the available evidence on FM in oncological populations. This gap limits the ability to define its true clinical relevance and to inform evidence-based, multidisciplinary management approaches. Therefore, a systematic review is warranted to synthesize current evidence, clarify the role of FM as comorbidity in cancer patients, and identify key areas for future research.

**Review objectives**

This review aims to systematically map the existing literature on FM in oncological patients, focusing on its prevalence, clinical characteristics, and impact on pain perception, Health-Related Quality of Life (HRQoL), and treatment adherence. Particular attention is given to FM as both a pre-existing condition and a comorbidity arising during or after cancer diagnosis and treatment, as well as to the diagnostic challenges in distinguishing FM-related pain from cancer-related pain. The review also examines the influence of FM on patient-reported outcomes, including symptom burden, functional impairment, and tolerance to oncological therapies. In addition, it provides an overview of current pharmacological and non-pharmacological management strategies in oncology settings, with a focus on lifestyle interventions such as physical activity and nutrition. By synthesizing the breadth of available evidence, this systematic review aims to identify key knowledge gaps and inform future research, ultimately supporting the development of integrated, multidisciplinary, and patient-centered approaches to the management of FM in cancer patients.

**Keywords**

Fibromyalgia; Cancer; Nociplastic pain; Quality of life; Integrated care; Nutrition; Physical activity; Pharmacology

**Country**

Italy

**ELIGIBILITY CRITERIA (PICO/PECO)**

**Population**

*Included:* Adult patients with fibromyalgia in the context of cancer or cancer-related pain

*Excluded:* Adults without fibromyalgia were excluded. Pediatric or adolescent populations were excluded. Patients with fibromyalgia not evaluated in the context of cancer or cancer-related pain were excluded. Animal and in vitro models were excluded.

**Intervention(s) or exposure(s)**

*Included:* The exposure of interest was fibromyalgia in the context of cancer or cancer-related pain. Eligible studies included observational (cross-sectional and cohort) and interventional designs involving adult patients with fibromyalgia within oncological populations. Studies were considered eligible if they investigated epidemiology, clinical characteristics, pain mechanisms, quality of life, or therapeutic management of fibromyalgia in cancer-related settings.

*Excluded:* Reviews, editorials, case reports, conference abstracts, and studies not specifically focusing on the relationship between fibromyalgia and cancer or cancer-related pain were excluded.

**Comparator(s) or control(s)**

This review does not have any comparators

**Study design**

Both randomized and nonrandomized study types will be included.

*Included:* Observational, cross-sectional, cohort, and interventional designs involving adult patients with FM in the context of cancer or cancer-related pain

*Excluded:* Reviews, editorials, case reports, conference abstracts, and studies not specifically focusing on the relationship between FM and cancer

**Context**

No specific contextual restrictions were applied.

**SEARCHING AND SCREENING**

**Search for unpublished studies**

Only published studies will be sought.

**Main sources that will be searched**

The main sources to be searched are *Embase*, *MEDLINE*, *PubMed* and *Scopus*.

**Search language restrictions**

The review will only include studies published in English.

**Search date restrictions**

Databases will be searched for articles published from 1 December 2023 and before by 30 November 2025.

**Selection process**

Studies will be screened independently by at least two people (or person/machine combination) with a process to resolve differences.

**DATA COLLECTION PROCESS**

**Data extraction from published articles and reports**

Data will be extracted independently by at least two people (or person/machine combination) with a process to resolve differences.

**Study risk of bias or quality assessment**

Risk of bias will be assessed using: *Cochrane RoB-2* and *ROBINS-I*

Additional information will be sought from study investigators if required information is unclear or unavailable in the study publications/reports.

**Reporting bias assessment**

For the assessment of risk of bias across the included studies, the ROBINS-I tool for observational studies and the RoB 2 tool randomized trial will be applied. For ROBINS-I, we will evaluated bias across seven domains: (1) confounding, (2) selection of participants, (3) classification of interventions, (4) deviations from intended interventions, (5) missing data, (6) measurement of outcomes, and (7) selection of the reported result. For RoB 2, the following domains will be considered: (1) bias arising from the randomization process, (2) bias due to deviations from intended interventions, (3) bias due to missing outcome data, (4) bias in measurement of the outcome, and (5) bias in selection of the reported result. The results will be summarized in a table using color-coded indicators to reflect the level of risk for each domain and each study, allowing a clear and rapid visualization of potential biases and supporting a transparent appraisal of the overall quality of evidence. The ROBVIS tool will be employed to generate graphical representations of the assessments, including traffic-light and summary plots, facilitating a visual comparison of risk across studies and domains.

**Certainty assessment**

Certainty of findings will not be assessed

**OUTCOMES TO BE ANALYSED**

**Main outcomes**

The synthesis of results will include a descriptive and thematic analysis of the available literature, organized according to study design, cancer type, patient population, fibromyalgia, diagnostic criteria, and treatment setting. Evidence on patient-reported outcomes, including symptom burden, functional impairment, psychological distress, and tolerance to oncological therapies, will be summarized and, where feasible, quantitatively tabulated. Findings will be synthesized to identify patterns, consistencies, and heterogeneity across studies, as well as key methodological limitations and gaps in the current literature.

**Additional outcomes**

The review will synthesize evidence on pharmacological and non-pharmacological management strategies for fibromyalgia in oncology settings through a structured descriptive approach. Interventions will be categorized according to type, including pharmacological treatments, multidisciplinary supportive care models, physical activity-based interventions and nutritional strategies. The synthesis will summarize the characteristics of the interventions, target populations, reported outcomes, and settings of care, with particular attention to their effects on pain management, symptom burden, functional status, Health-Related Quality of Life and treatment tolerance.

**Full Search Query**

(("fibromyalgia"[MeSH Terms] OR "fibromyalgia"[All Fields] OR "fibromyalgias"[All Fields])

AND

("cancer s"[All Fields] OR "cancerated"[All Fields] OR "canceration"[All Fields] OR "cancerization"[All Fields] OR "cancerized"[All Fields] OR "cancerous"[All Fields] OR "neoplasms"[MeSH Terms] OR "neoplasms"[All Fields] OR "cancer"[All Fields] OR "cancers"[All Fields]))

AND

((y_10[Filter]) AND (casereports[Filter] OR clinicalstudy[Filter] OR clinicaltrial[Filter] OR comparativestudy[Filter] OR controlledclinicaltrial[Filter] OR multicenterstudy[Filter] OR observationalstudy[Filter] OR randomizedcontrolledtrial[Filter])))

**MeSH/Field Terms**

| Concept | MeSH / Field Terms |
| --- | --- |
| Fibromyalgia | "fibromyalgia"[MeSH Terms] OR "fibromyalgia"[All Fields] OR "fibromyalgias"[All Fields] |
| Cancer | "cancer's"[All Fields] OR "cancerated"[All Fields] OR "canceration"[All Fields] OR "cancerization"[All Fields] OR "cancerized"[All Fields] OR "cancerous"[All Fields] OR "neoplasms"[MeSH Terms] OR "neoplasms"[All Fields] OR "cancer"[All Fields] OR "cancers"[All Fields] |

The protocol will be registered on OSF.
