# Supplementary material for: Fibromyalgia in cancer patients: a systematic review and clinical implications for integrated care
Source: Front Pain Res (Lausanne). 2026 Jun 11;7:1851474. doi: 10.3389/fpain.2026.1851474 (PMC13294063; doi:10.3389/fpain.2026.1851474)
Supplement: Supplementary file 4 [file Table4.docx]

Supplementary Material

[1 Supplementary Tables 2](#_Toc226626024)

[1.1 Table S4. Characteristics and main findings of studies (n=11) 2](#_Toc226626025)

[2 Supplementary data 5](#_Toc226626027)

[2.1 Full Search Query 5](#_Toc226626028)

[2.2 Translation of Terms 5](#_Toc226626029)

# Supplementary Tables

## Table S4. Characteristics and main findings of studies (n=11)

| **Study** | **Aim** | **Design** | **N** | **Sex** | **Age** | **Methods** | **Key results** |
| --- | --- | --- | --- | --- | --- | --- | --- |
| Savin E. et al., 2022 [26] | To examine the association between FM and benign and malignant GI disorders | Retrospective cross-sectional study | 18,598 | M/F | 56.5 ± 14.0 | Comparative analysis of gastrointestinal comorbidities in FM patients and matched controls | No significant difference in the prevalence of gastrointestinal malignancies between FM patients and controls |
| Wolfe F. et al., 2020 [38] | To evaluate the association between FM, widespread pain, and cancer-related mortality | Observational cohort study | 35,248 | M/F | 62.1 ± 13.7 | Cox proportional hazards models with long-term mortality follow-up | No evidence of an association between FM or widespread pain and cancer mortality |
| Joyce E. et al., 2023 [39] | To assess the relationship between baseline nociplastic pain and discontinuation of adjuvant endocrine therapy | Retrospective single-center cohort study | 681 | F | NR | Univariate and multivariable Cox proportional hazards regression | Pre-existing nociplastic pain was associated with premature discontinuation of endocrine therapy; FM screening scores may identify patients at higher risk |
| Mancuso A.C. et al., 2020 [40] | To assess associations between infertility, HRQoL, and medical comorbidities in women veterans | Cross-sectional observational study | 996 | F | 39.9 ± 7.8 | Multivariate regression analyses with age-adjusted models | History of infertility was associated with poorer physical HRQoL and increased prevalence of FM |
| Üçüncü M.Z. et al., 2020 [41] | To evaluate sociodemographic characteristics of IBS patients and the association between IBS and FM | Observational study | 202 | M/F | FM: 38.8 ± 11.3; non-FM: 38.5 ± 12.2 | Group comparisons and multivariate logistic regression with ROC analysis | FM was frequently observed among IBS patients and was associated with specific clinical and sociodemographic characteristics |
| Gatta et al., 2021 [42] | To investigate the association between pain experienced during mammographic screening and FM diagnosis | Prospective observational study | 1,060 | F | 54 | Ordinal logistic regression models (univariate and multivariate) | High pain intensity during mammography was associated with FM; 36% of women reporting severe pain had an FM diagnosis |
| Fan et al., 2022 [43] | To investigate diagnosis, treatment, and satisfaction in individuals with pain conditions using real-world data | Retrospective cross-sectional study | 64,991 | M/F | Cancer pain: 50.7 ± 18.4; FM: 44.5 ± 15.3 | Analysis of China National Health and Wellness Survey using t-tests and chi-square tests | Treatment satisfaction was lowest among patients with cancer-related pain; awareness and diagnosis of FM and cancer-associated pain remained suboptimal |
| Aviles Gonzalez et al., 2022 [44] | To assess HRQoL in patients with solid cancer and the role of depressive episodes | Cross-sectional study | 151 | M/F | 63.1 ± 11.5 | One-way ANOVA controlling for cancer stage | The negative impact of FM on HRQoL was significantly greater than that attributable to solid cancer |
| Figueroa-Parra G. et al., 2023 [45] | To assess the prevalence and predictors of long-term opioid therapy in patients with SLE | Cross-sectional study | 465 | M/F | 53.1 ± 16.2 | Logistic regression analyses of demographic and clinical predictors | FM was strongly associated with long-term opioid therapy use among patients with SLE |
| Alkabbani W. et al., 2019 [46] | To estimate persistence of prescribed cannabinoid use and associated predictors | Retrospective cohort study | 5,452 | M/F | 51.9 ± 15.0 | Kaplan–Meier survival analysis and competing-risk regression models | Cancer was associated with higher discontinuation of cannabinoids, whereas FM was associated with greater treatment persistence |
| Bozorgi H. et al., 2021[47] | To evaluate the efficacy of crocin for CIPN | Double-blind, placebo-controlled crossover trial | 253 | M/F | 61.5 ± 3.6 | Randomized crossover intervention with weekly outcome assessments | Crocin significantly improved CIPN symptoms compared with placebo, supporting its potential role in cancer-related pain management |
| M= Male; F= Female; FM= Fibromyalgia; HRQoL= Health-Related Quality Of Life; SLE= systemic lupus erythematosus; IBS= Irritable Bowel Syndrome; CIPN= chemotherapy-induced peripheral neuropathy; GI= Gastrointestinal. | | | | | | | |

# Supplementary data

## Full Search Query

(("fibromyalgia"[MeSH Terms] OR "fibromyalgia"[All Fields] OR "fibromyalgias"[All Fields])

AND

("cancer s"[All Fields] OR "cancerated"[All Fields] OR "canceration"[All Fields] OR "cancerization"[All Fields] OR "cancerized"[All Fields] OR "cancerous"[All Fields] OR "neoplasms"[MeSH Terms] OR "neoplasms"[All Fields] OR "cancer"[All Fields] OR "cancers"[All Fields]))

AND

((y_10[Filter]) AND (casereports[Filter] OR clinicalstudy[Filter] OR clinicaltrial[Filter] OR comparativestudy[Filter] OR controlledclinicaltrial[Filter] OR multicenterstudy[Filter] OR observationalstudy[Filter] OR randomizedcontrolledtrial[Filter])))

## Translation of Terms

| Concept | MeSH / Field Terms |
| --- | --- |
| Fibromyalgia | "fibromyalgia"[MeSH Terms] OR "fibromyalgia"[All Fields] OR "fibromyalgias"[All Fields] |
| Cancer | "cancer's"[All Fields] OR "cancerated"[All Fields] OR "canceration"[All Fields] OR "cancerization"[All Fields] OR "cancerized"[All Fields] OR "cancerous"[All Fields] OR "neoplasms"[MeSH Terms] OR "neoplasms"[All Fields] OR "cancer"[All Fields] OR "cancers"[All Fields] |
