# Supplementary material for: Fibromyalgia in cancer patients: a systematic review and clinical implications for integrated care
Source: Front Pain Res (Lausanne). 2026 Jun 11;7:1851474. doi: 10.3389/fpain.2026.1851474 (PMC13294063; doi:10.3389/fpain.2026.1851474)
Supplement: Supplementary file 5 [file Table5.docx]

**Table S5.** Main tools adopted for fibromyalgia assessment. Indication of the main questionnaires used to evaluate fibromyalgia, including FIQ/FIQ-R, PROMIS, EORTC QLQ-C30, SF-36/SF-12, and MDASI.

| **Instrument** | **Target population** | **Main domains** | **Usage notes** |
| --- | --- | --- | --- |
| FIQ / FIQ-R [121] | FM | Pain, fatigue, stiffness, physical function, emotional status | Longitudinal monitoring of FM symptoms |
| PROMIS [122] | Chronic / oncology patients | Pain, fatigue, sleep, anxiety, depression, social participation | Flexible, allows comparison between populations, continuous monitoring |
| EORTC QLQ-C30 [119] | Oncology patients | Physical function, social role, emotional well-being, cancer-related symptoms | Widely validated; can be combined with tumor-specific modules |
| SF-36 / SF-12 [123,124] | General/chronic patients | Physical function, pain, vitality, mental well-being, social function | Generic tool; useful for multidimensional assessments |
| MDASI [125] | Oncology patients | Symptom severity and interference (pain, fatigue, nausea, anxiety) | Useful to monitor treatment-related symptoms |
| FIQ/FIQ-R = FM Impact Questionnaire / Revised version; PROMIS = Patient-Reported Outcomes Measurement Information System; EORTC QLQ-C30 = European Organisation for Research and Treatment of Cancer QoL Questionnaire – Core 30; SF-36 / SF-12 = Short Form Health Survey 36 / 12; MDASI = MD Anderson Symptom Inventory. | | | |
